# Chloroplast phylogenomic analysis provides insights into the evolution of the largest eukaryotic genome holder, *Paris japonica* (Melanthiaceae)

**DOI:** 10.1186/s12870-019-1879-7

**Published:** 2019-07-04

**Authors:** Lifang Yang, Zhenyan Yang, Changkun Liu, Zhengshan He, Zhirong Zhang, Jing Yang, Haiyang Liu, Junbo Yang, Yunheng Ji

**Affiliations:** 1 Key Laboratory for Plant Diversity and Biogeography of East Asia, Kunming Institute of Botany, Chinese Academy of Sciences, Kunming, Yunnan People’s Republic of China; 2 Yunnan Key Laboratory for Integrative Conservation of Plant Species with Extremely Small Population, Kunming Institute of Botany, Chinese Academy of Sciences, Kunming, Yunnan China; 3 Germplasm Bank of Wild Species, Kunming Institute of Botany, Chinese Academy of Sciences, Kunming, Yunnan China; 4 State Key Laboratory of Phytochemistry and Plant Resources in West China, Kunming Institute of Botany, Chinese Academy of Sciences, Kunming, Yunnan China; 5 School of Life Science, Yunnan University, Kunming, China

**Keywords:** Plastome, Phylogenomics, Giant genome, Evolution, *Paris japonica*, *Paris*, *Trillium govanianum*

## Abstract

**Background:**

Robust phylogenies for species with giant genomes and closely related taxa can build evolutionary frameworks for investigating the origin and evolution of these genomic gigantisms. *Paris japonica* (Melanthiaceae) has the largest genome that has been confirmed in eukaryotes to date; however, its phylogenetic position remains unresolved. As a result, the evolutionary history of the genomic gigantisms in *P. japonica* remains poorly understood.

**Results:**

We used next-generation sequencing to generate complete plastomes of *P. japonica*, *P. verticillata*, *Trillium govanianum*, *Ypsilandra thibetica* and *Y. yunnanensis*. Together with published plastomes, the infra-familial relationships in Melanthiaceae and infra-generic phylogeny in *Paris* were investigated, and their divergence times were calculated. The results indicated that the expansion of the ancestral genome of extant *Paris* and *Trillium* occurred approximately from 59.16 Mya to 38.21 Mya. The sister relationship between *P. japonica* and the section *Euthyra* was recovered, and they diverged around the transition of the Oligocene/Miocene (20 Mya), when the Japan Islands were separated from the continent of Asia.

**Conclusions:**

The genome size expansion in the most recent common ancestor for *Paris* and *Trillium* was most possibly a gradual process that lasted for approximately 20 million years. The divergence of *P. japonica* (section *Kinugasa*) and other taxa with thick rhizome may have been triggered by the isolation of the Japan Islands from the continent of Asia. This long-term separation, since the Oligocene/Miocene boundary, would have played an important role in the formation and evolution of the genomic gigantism in *P. japonica.* Moreover, our results support the taxonomic treatment of *Paris* as a genus rather than dividing it into three genera, but do not support the recognition of *T. govanianum* as the separate genus *Trillidium*.

**Electronic supplementary material:**

The online version of this article (10.1186/s12870-019-1879-7) contains supplementary material, which is available to authorized users.

## Background

Angiosperms exhibit extreme diversity in genome size that is defined as the haploid nuclear DNA amount, varying by approximately 2400-fold between the smallest and largest genomes [1–4]. Although the distribution of genome size in angiosperms is strongly skewed towards small genomes (with a mean value of 1C = 5.7 Gb and a modal value of 1C = 0.6 Gb) [4], to date, five species with the genome size 1C > 100 Gb have been documented. These plant species belong to the monocotyledonous family Melanthiaceae (one species in *Paris*, two species in *Trillium*), Liliaceae (one species in *Fritillaria*), and eudicot family Viscaceae (one species in *Viscum*) [5–9], suggesting that genomic gigantism may have originated and evolved independently in only a few lineages [1, 10].

Because of the technical challenges in sequencing very large or very small genomes, insights into the mechanisms that drive the formation of genomic gigantism remain limited [9]. High-resolution and well-supported phylogenetic relationships between species with giant genomes and their closely related taxa can build evolutionary frameworks to elucidate the evolutionary history of these genomic gigantisms [9–12]. Unfortunately, a robust phylogeny for the genera *Paris*, *Trillium* and *Viscum*, which include genomic gigantisms, remains elusive [13–15], which impedes our understanding of the mechanisms underlying the formation and evolution of giant genomes.

Although the genome size of *Polychaos dubia*, a unicellular eukaryote, has been estimated to be over 670 Gb [16], this measurement is considered unreliable and inaccurate [4]. To date, the confirmed largest genome in eukaryotes has been observed in *Paris japonica* (Franch. et Sav.) Franch. (also known as *Kinugasa japonica* (Franch. et Sav.) Tatew.et Sutô.), with the 1C value of 148.88 Gb [1, 17]. This plant is a perennial herb belonging to the monocotyledonous family Melanthiaceae tribe Parideae [18, 19], and occurs natively in central and northern Honshu, Japan [20, 21]. Cytological studies revealed that *P. japonica* is an octoploid with a chromosome number of 2n = 8x = 40 [1, 22, 23]. Because of its distinctive characters, such as showy and white sepals, and octoploid chromosome count, *P. japonica* has been historically placed either in the genus *Paris* (section *Kinugasa*) [21, 23] or treated as a monotypic genus *Kinugasa* [20, 24]. Moreover, the evolutionary relationships of *P. japonica* with related taxa have remained controversial in recent analyses based on single or multiple-locus DNA sequences. An analysis using the plastid *rbcL* region indicated that *P. japonica* is a sister to the genus *Trillium* [25]. A combination analyses of the plastid *rbcL* and *matK* and nuclear ITS DNA regions revealed that *P. japonica* is closely related to the genus *Daiswa* (=*Paris* section *Euthyra*) [13, 26]. By contrast, two independent studies that based on the plastid *psbA-trnH*, *trnL*-*F* and nuclear ITS sequence data [27], and the combination of five plastid regions (*atpB*, *rbcL*, *matK*, *ndhF* and *trnL-F*) [28], resolved *P. japonica* as the sister group of the section *Paris*. These conflicts suggest that the relationships between *P. japonica* and allied taxa require further investigation.

Phylogenetic analysis using too few DNA sequences may result in a conflict between different sequence regions [29, 30]; in such a case, it is not possible to reconstruct a robust and reliable phylogeny, in particular, at low taxonomic levels [31]. Because of its high level of intra- and infra-specific sequence variation, complete plastome DNA sequeces can offer valuable information for the analysis of complex evolutionary relationships in plants [32–34]. With the advent of next-generation DNA sequencing technologies, plasotmes have been widely used in recent years to reconstruct robust phylogenies for several phylogenetically difficult plant taxa [31, 35–37]; these cases suggest that whole plastome sequencing may provide novel evidence to elucidate the relationships between *P. japonica* and allied taxa. Despite the fact that the analysis of maternally inherited DNA loci may not demonstrate the complete history of the species, the complete plastome-based phylogeny can give us some valuable information to elucidate the maternal origin and evolution of the genomic gigantisms in *P. japonica.*

In the current study, we used low-coverage genome shotgun sequencing [38] to generate plastomes of *P. japonica*, *P. verticillata*, *Trillium govanianum*, *Ypsilandra thibetica* and *Y. yunnanensis* and then inferred the molecular evolution by comparing the structure and gene content to those of other published plastomes in Melanthiaceae. Then, we reconstructed the evolutionary relationships within the family to investigate the phylogenetic position of *P. japonica*. Finally, we dated the divergence of *P. japonica* to provide insights into the evolutionary history of the largest eukaryotic genome holder.

## Results

### Plastid genome features

The plastome of *P. japonica*, *P. verticillata*, *T. govanianum*, *Y. thibetica* and *Y. yunnanensis* were completely assembled. The sequencing coverage for each plastome ranged from 283× to 1086× (Additional file 2: Table S2). The gene content (Additional file 3: Table S3, Additional file 4: Table S4, Additional file 5: Table S5, Additional file 6: S6, Additional file 7: S7) and arrangement (Additional file 8: Figure S1, Additional file 9: Figure S2, Additional file 10: Figure S3, Additional file 11: Figure S4, Additional file 12: Figure S5) across the five plastomes were almost identical. The size of these newly generated plastomes ranged from 155,957 to 158,806 bp, which exhibited a typical quadripartite structure with a pair of IRs (26,805–27,602 bp) separated by the LSC (83,635–85,301 bp) and SSC (18,337–19,586 bp) regions (Table 1). Except for the *trnD-GUC* that has been deleted from the plastome of *Y. thibitica*, the other plastomes encoded 114 unique genes, including 80 protein-coding genes, 30 tRNA genes, and 4 rRNA genes (Table 2).

**Table 1:** Size of plastomes reported in this study

Species	Whole plastome size	LSC size	SSC size	IR size
*Paris japonica*	155,957 bp	83,635 bp	18,712 bp	26,805 bp
*P. verticillata*	157,946 bp	83,710 bp	19,586 bp	27,325 bp
*Trillium govanianum*	157,379 bp	83,802 bp	18,651 bp	27,463 bp
*Ypsilandra yunnanensis*	158,806 bp	85,301 bp	18,383 bp	27,561 bp
*Y. thibetica*	157,613 bp	84,072 bp	18,337 bp	27,602 bp

**Table 2:** Summary of gene content in the five newly sequenced plastomes

Species	No. of protein-coding genes	No. of tRNA	No. of rRNA	Total
*Paris japonica*	80	30	4	114
*P. verticillata*	80	30	4	114
*Trillium govanianum*	80	30	4	114
*Ypsilandra yunnanensis*	80	30	4	114
*Y. thibetica*	80	29	4	113

**Fig. 1:**
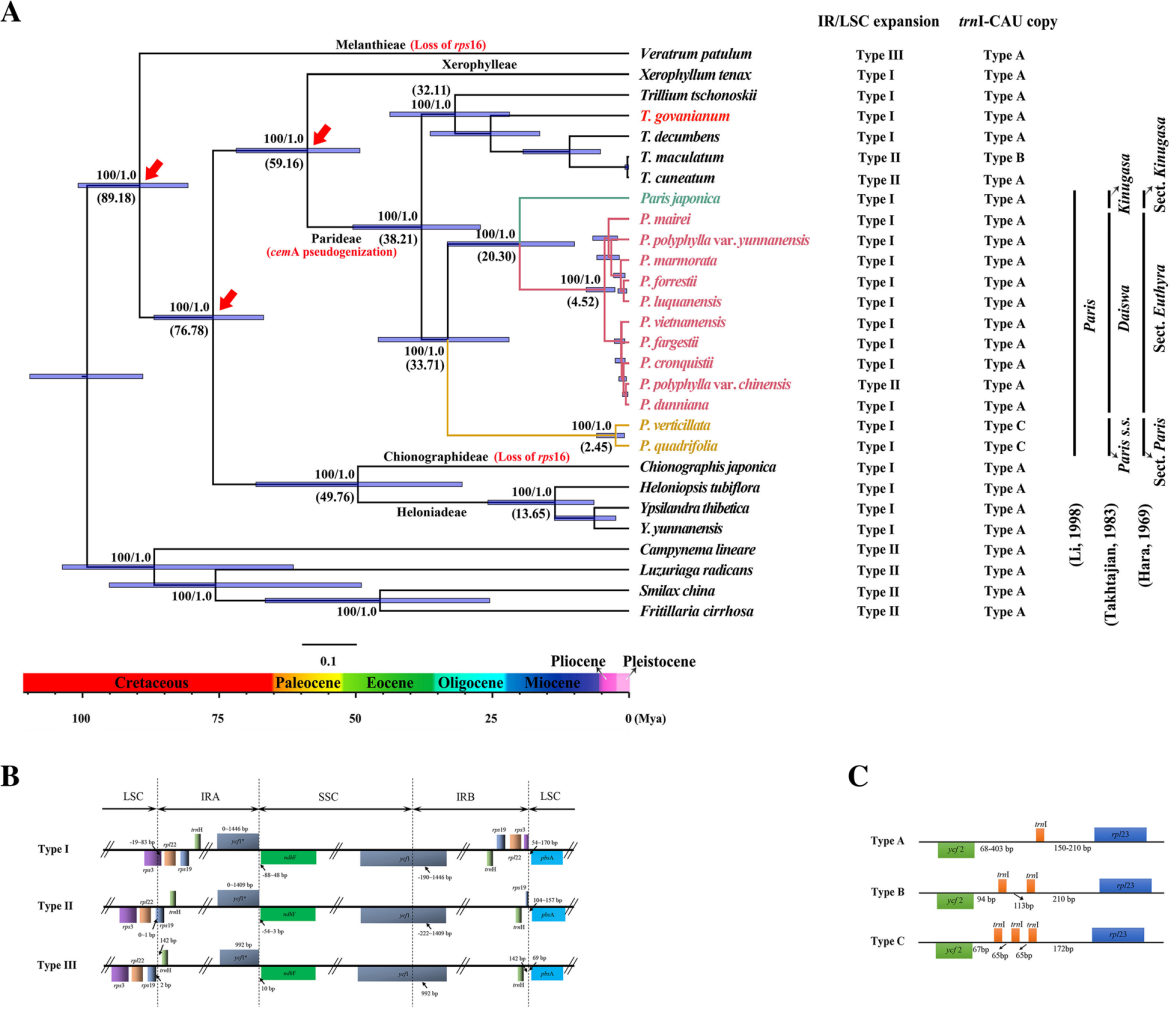
Phylogeny, molecular dating, comparison of IR expansions and *trn*I-CAU copy number based on complete plastome DNA sequences from Melanthiaceae. **a** Phylogenetic relationships within Melanthiaceae based on complete cp genome sequences. Patterns of IR expansion and copy number of *trn*L-CAU for each species were mapped along the tree. Numbers above/under the tree branches represent BS and PP values/(mean divergent ages). Arrows indicated the calibrating points for molecular dating. Horizontal blue bars on each node indicate the 95% confidence interval of divergence time. Numbers on the Time Axis indicate million year ago (Mya). **b** Three types of IR/LSC expansions were detected in the plastomes within Melanthiaceae. **c** Single-copies, duplicates, and triplicates of the *trn*I-CAU gene were found in the plastomes within Melanthiaceae

Although the gene content and arrangement were almost identical, pseudogenization and gene loss were found to have occasionally occurred within the family Melanthiaceae. Because of the presence of several internal stop codons in coding regions, *cemA* was identified as a pseudogene in all *Paris* and *Trillium* plastomes (Fig. 1a). In addition, the loss of the first exon of *rps*16 gene was found in the plastomes of *Veratrum patulum* and *Chionographis japonica* (Fig. 1a). Expansion of the IR regions into the *ycf*1 gene at the IR/SSC boundary occurred identically in all plastomes in Melanthiaceae, whereas their IR/LSC junctions were significantly variable. Three types of IR/LSC boundaries were observed in Melanthiaceae and outgroup taxa (Fig. 1b). The expansion of IR into the *trnH-rps19* intergenic spacer (type III) was only found in *V. patulum*, whereas the expansion of IR into *rps19* (type II) occurred in *Trillium cuneatum*, *T. maculatum,* and *Paris polyphylla* var. *chinensis*, as well as in outgroup taxa. Comparatively, characterized by the IR/LSC boundary falling into *rps3*, type I was observed in the remaining taxa (Fig. 1a).

The length of the intergenic region between *rpl*23 and *ycf*2 exhibited substantial variation among plastomes in the family Melanthiaceae, within which single-copy, duplicates and triplicates of *trn*I-CAU were observed (Fig. 1c). Triplication of *trn*I-CAU (type C) was observed in *P. quadrifolia* and *P. verticillata* (section *Paris*), whereas duplication of *trn*I-CAU (type B) was found in *T. maculatum*. A single-copy of *trn*I-CAU (type A) was identified in the other plastomes (Fig. 1a).

### Phylogenomic analysis and divergence estimation

The tree topologies from both ML and BI analyses were identical. The phylogenetic relationships among the plastomes are presented in Fig. 1a. Five well-supported clades (BS = 100%, PP =1), corresponding to the five tribes (Melanthieae, Chionographideae, Heloniadeae, Xerophylleae, and Parideae) recognized by Zomlefer [18], were recovered. The tribe Melanthieae was sister to the rest of Melanthiaceae (BS = 100%, PP =1). The sister relationships between Chionographideae and Heloniadeae, as well as between Xerophylleae and Parideae, were fully supported (BS = 100%, PP =1). The intra-tribe relationships from our phylogenomic analysis are congruent with those of previous studies based on the nuclear ribosomal ITS and plastid *trn*L-*trn*F regions [18]; the combination of plastid DNA sequences [28, 39]; and the plastid genome sequencing [15].

Within the tribe Parideae, the sister relationship between *Trillium* and *Paris* was recovered (BS = 100%, PP =1). The *Paris* species were further grouped into three fully supported lineages (BS = 100%, PP =1) that correspond to either the three narrowly-defined genera (*Paris s.s.*, *Kinugas* and *Daiswa*, respectively) by Takhtajan [24] or the three sections (section *Paris*, section *Kinugasa* and section *Euthyra,* respectively) circumscribed by Hara [23]. Among them, *P. japonica* (section *Kinugasa*) was sister to the section *Euthyra* (BS = 100%, PP =1), and the section *Paris* was sister to the clade consisting of section *Kinugasa* and section *Euthyra*. The intersectional relationships obtained here are consistent with those of a previous study [40].

Three calibration points in Melanthiaceae (Fig. 1a) suggested by previous study [41] were used to constrain the plastome-based phylogenetic tree. The results suggested that the most recent common ancestor (MRCA) for the tribe Parideae dated at approximately 59.16 Mya (95% HPD: 73.01–49.11 Mya) and the genera *Paris* and *Trillium* diverged from each other approximately 38.21 Mya (95% HPD: 52.17–26.84 Mya). Within the genus *Paris*, the MRCA of the section *Paris* dated at approximately 33.71 Mya (95% HPD: 47.47–22.03 Mya), and the divergence between the monotypic section *Kinugasa* (*P. japonica*) and the section *Euthyra* occurred approximately 20.30 Mya (95% HPD: 34.64–9.96 Mya).

## Discussion

Robust phylogenies for species with giant genomes and allied taxa can build evolutionary frameworks to elucidate the origin and evolution of these genomic gigantisms [9–12]. Previous studies [13, 25–27] revealed that it is difficult to reconstruct high-resolution and well-supported phylogenetic relationships between *P. japonica*, the largest eukaryotic genome holder, and its allied taxa based on too few DNA sequence regions. In this study, we sequenced the whole plastomes of *P. japonica*, as well as *P. verticillata*, *Trillium govanianum*, *Ypsilandra thibetica* and *Y. yunnanensis*. Coupled with publicly available plastomes in Melanthiaceae, we performed comparative and phylogenetic analyses of whole plastomes to clarify the evolutionary relationships of *P. japonica* with its closely related taxa. This study gives us some new information about the origin and evolution of the genomic gigantisms in *P. japonica.*

### Plastome comparison

The loss of the first exon of *rps16* was observed in the phylogenetically distinctive tribes Melanthieae and Chionographideae. Furthermore, the loss of *trn*D-GUC was only found in *Y. thibitica.* These results support the deduction that the loss of certain plastid genes may have independently occurred over the evolutionary history of angiosperms [32, 42]. Therefore, the loss of certain plastid genes may not provide relevant evolutionary information. However, neither gene loss nor gene relocation were observed in any of the Melanthiaceae plastomes, implying the gene content and plastome structure in the family are highly conserved.

Previous studies have revealed that the protein-coding gene *cemA* has been lost in several non-photosynthetic parasitic plants [43–45]. To our knowledge, pseudogenization of this gene in photosynthetic autotrophic angiosperms has been only detected in the closely related genera *Paris* and *Trillium* (Fig. 1a). Although its function remains unclear [46], this mutation may provide a molecular synapomorphy to recognize the tribe Parideae [47]. In addition, as proposed in a previous study [15], the lineage-specific triplication of *trnI-CAU* in *P. quadrifolia* and *P. verticillata* could be used as a molecular synapomorphy to circumscribe the section *Paris* (Fig. 1a).

The IR/LSC boundaries of monocot plastoms generally expand into the *trnH*-*rps19* gene cluster and the IR expansion duplicate *trnH* gene, which differs from those of non-monocot angiosperms [47]. In this study, we identified three types of IR/LSC expansions within Melanthiaceae; of those, type II and III exhibited the typical monocot IR/LSC junctions, whereas the IR/LSC junctions of type I fell in *rps3*. Although IR/LSC expansions into the *rps19*-*rpl22* intergenic spacer or *rpl22* have been observed in some monocot orders, such as Asparagales, Commelinales, Zinbiberales and Poales [48–50], the more progressive expansion of IR/LSC into *rps3* has only been found in Melanthiaceae to date. The phylogenetic distribution of the three types of IR/LSC boundary in the tree topology suggests that the type III can be the ancestral state in Melanthiaceae, by compared with the expansion of IR regions into *rps3* occurring in the derived tribes such as Chionographideae, Heloniadeae, Xerophylleae, and Parideae (Fig. 1a). Furthermore, the observation of type II of IR/LSC junction in *T. cuneatum*, *T. maculatum,* and *Paris polyphylla* var. *chinensis* may have been resulted from a secondary slippage of IR regions from *rps3* to *rps19*.

### Phylogeny inferences

Our phylogenomic analysis recovered five well-supported lineages (BS = 100%, PP = 1) within Melanthiaceae, which correspond to the five tribes recognized by Zomlefer [18]. The evolutionary relationships recovered in this study are consistent with those of previous investigations [18, 28, 40, 51] but with higher branch support (BS = 100%, PP = 1). The results further justify that whole plastid genome sequencing can improve the phylogenetic resolution in a certain lineage [33, 34].

Our expanded sampling of the plastomes in Parideae provided an opportunity to reconstruct a robust intra-generic phylogeny in the tribe. The basal divergence in Parideae occurred approximately 38.21 Mya, forming two fully supported lineages (*Paris* and *Trillium*) in the tree topology (BS = 100%, PP = 1). The two genera share synapomorphies, including a single whorl of net-veined leaves presenting at a stem apex, a stem apex bearing a solitary flower, and a chromosome base number *n* = 5 [16]. Within the clade *Paris*, the three sections (section *Paris*, section *Kinugasa*, and section *Euthyra*) outlined by Hara [23] as well as the three narrowly defined genera *Paris s.s.*, *Daiwa* and *Kinugasa* by Takhtajan [24] were each recovered as monophyletic clades with strong support (BS = 100%, PP = 1) in both the ML and BI analyses. Given that species in the *Paris* clade share the morphological synapomorphies of flowers and leaves, 4- to 15-merous compared with the trimerous condition of *Trillium* [27], we correspondingly prefer to accept the taxonomic treatment of *Paris* as a single genus [21, 23] rather than in three separated genera [24].

**Fig. 2:**
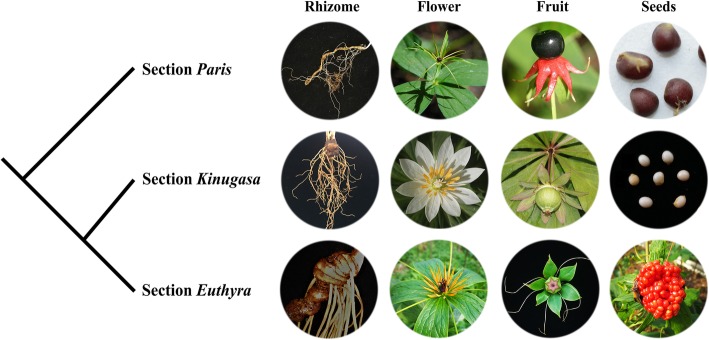
Comparison of morphological features among *Paris japonica* (section *Kinugasa*), section *Paris,* and section *Euthyra*

Since a previous study had not included the plastome of *P. japonica* in its phylogenetic analysis, its evolutionary relationships with other *Paris* species remained unresolved [15]. Both ML and BI analysis identically indicated that *P. japonica* (section *Kinugasa*) is a sister to the section *Euthyra*, which is congruent with the analyses of the plastid *rbcL*, *matK* and *trnL-trnF* regions [13, 26, 40]. However, the relationships recovered by our data largely differ from the results of combination analysis of plastid *psb*A*-trn*H and *trn*L*-*F and nuclear ITS sequences [27], and plastid *atpB*, *rbcL*, *matK*, *ndhF* and *trnL-F* regions [28]. It is noteworthy that, the well-supported sister relationship between *P. japonica* and the section *Euthyra* (BS = 100%, PP = 1) recovered in this study, can be also justified by the morphological synapomorphies that they share, such as a thick rhizome and angular ovary, in contrast to the long and slender rhizome and rounded ovary species of the section *Paris* (Fig. 2). In addition, the unusual morphological characteristics of the species (i.e., the showy, white sepals, and octoploid chromosome number) justify the taxonomic treatment of *P. japonica* as a distinctive section within the genus *Paris* by Hara [23].

**Fig. 3:**
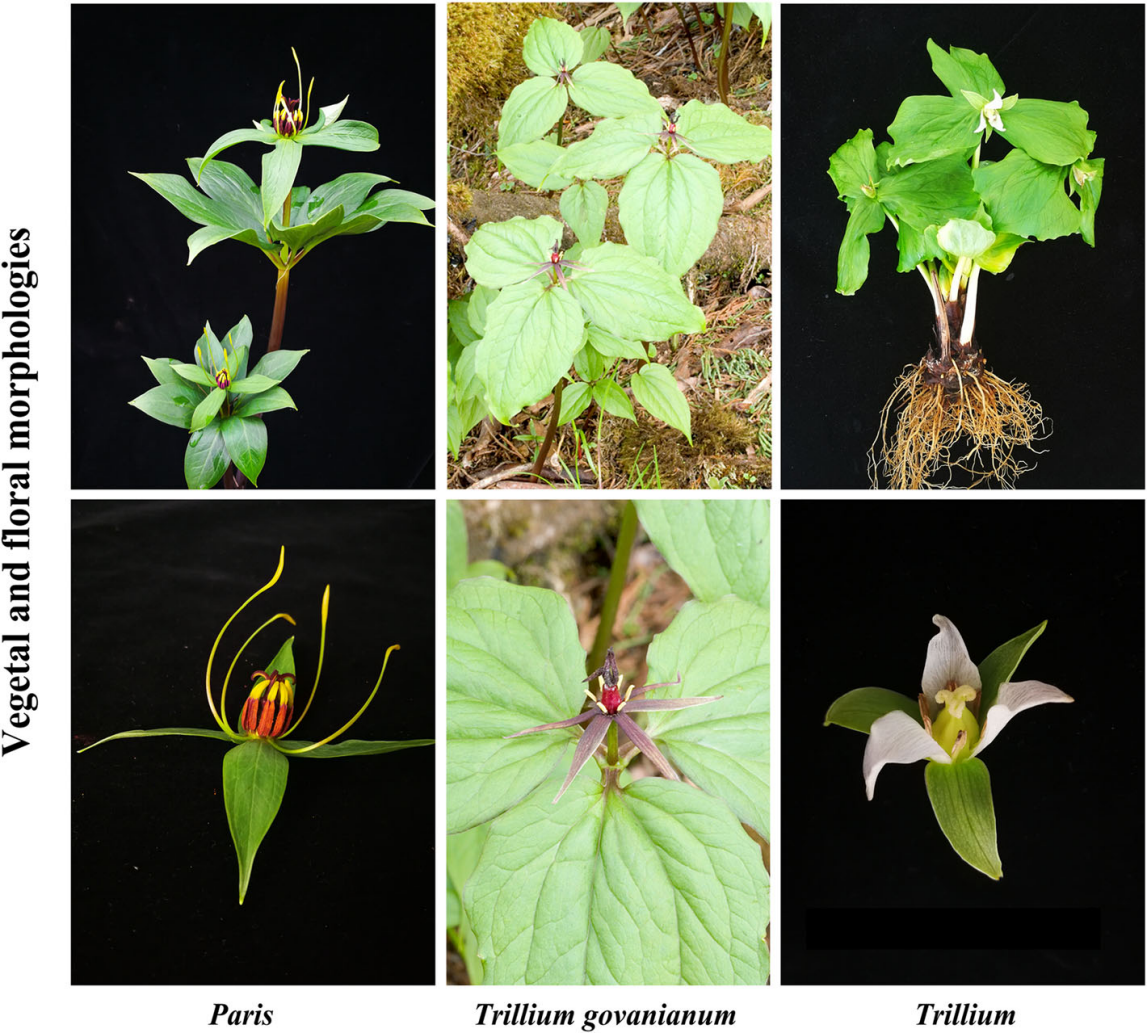
Comparison of vegetal and floral morphologies among *Trillium govanianum*, *Paris,* and typical *Trillium* species

Our data not only recovered the evolutionary backbone in *Paris* but also offered evidence to clarify disputes about the phylogenetic position of *T. govanianum*, which occurs natively in the Himalayan mountains. Although *T. govanianum* has a trimerous flower and leaves like those of *Trillium* species, it shares morphological features, such as narrow sepals and filiform petals, with *Paris* species (Fig. 3). Accordingly, *T. govanianum* was recognized as a separate genus *Trillidium* [13, 52]. However, neither the ML nor BI tree topology separated *T. govanianum* from the *Trillium* species but grouped them into a well-supported clade (BS = 100%, PP = 1). It is notable that similar finding has been shown in the phylogenetic analysis based on five plastid DNA regions that has a more extensive taxon sampling of Melanthiaceae [28]. Taken together, the results suggest that *T. govanianum* should remain in the genus *Trillium* and deny the recognition of the genus *Trillidium*.

### Insights into the origin and evolution of the genomic gigantism in *Paris japonica*

The robust phylogeny reconstructed in the current study provided insights into the origin and evolution of the genomic gigantism in *P. japonica*. Most species in Melanthiaceae possess small or very small genomes, while large or giant genomes have been exclusively found in the two genera: *Paris* and *Trillium* [40]. Character reconstruction revealed that a genome size increase (more than four-fold) possibly occurred after the divergence of Xerophylleae and Parideae, but before the differentiation between *Paris* and *Trillium* [40]. Molecular dating indicated that the stem age and crown age of Parideae were approximately 59.16 Mya and 38.21 Mya, respectively, suggesting that the massive genome expansion would have lasted for a long period of approximately 20 million years. During this period, the ancestral genome of extant *Paris* and *Trillium* would have gradually expanded, implying that the genome size increase in Parideae could be the slow accumulation over tens of millions of years as a previous study proposed [40].

The phylogenomic analyses indicated that the section *Paris* is sister to the clade including *P. japonica* (section *Kinugasa*) and the section *Euthyra*. The relationships suggest that the formation of a giant genome in *P. japonica* most likely took place after the divergence of the sections *Euthyra* and *P. japonica*. Except for *P. japonica*, two species (*T.* × *hagae* and *T. rhombilolium*) with genome sizes 1C > 100 Gb have been found in the genus *Trillium* [5, 6, 9]. As we did not obtain samples of these two plants, their phylogenetic positions within *Trillium* remain unclear. Nevertheless, the evolutionary relationships of *P. japonica* with related taxa recovered in the study reveal that the formation of the giant genomes in *P. japonica* and *Trillium* species may have been independent events.

The coalescence of the plastomes of *P. japonica* and the section *Euthyra* occurred around the transition of the Oligocene/Miocene (20.30 Mya, 95% HPD: 34.64–9.96 Mya), when the opening of the Japan Sea separated the Japan Islands from the continent of Asia [53]. Although *P. japonica* and the section *Euthyra* are closely related, they occupy distinct distributions: *P. japonica* is endemic to Japan, whereas species from the section *Euthyra* are chiefly distributed in subtropical China and the Himalayas [23]. Hence, the divergence of *P. japonica* and the section *Euthyra* may have been triggered by the isolation of the Japan Islands from the continent of Asia.

Notably, the genome size of *P. japonica* is approximately 2–3 folds larger than that of those species belonging to the section *Euthyra* [40]. A line of evidence justifies that the genome size variation in plants is under selective constrains and has not evolved by a pure drift process [54–56]. As a result, genome size can be strictly related to the environment and ecology of a species [57]. In general, plants with larger genomes share some morphological traits, such as large body and stomata size [58]. Due to the drought susceptibility of the plants with large stomata, only those species occurring in humid habitats can sustain larger genomes [56, 58]. Compared with the monsoonal climate which is characterized by obvious precipitation seasonality in subtropical China and the Himalayas [59, 60], the maritime climate of the Japan islands [61] would create relatively more humid habitats that facilitate the evolution of *P. japonica* toward genomic gigantism.

## Conclusions

The evolutionary relationships of the largest eukaryotic genome holder, *P. japonica*, with its closely related taxa were investigated by comparative and phylogenetic analyses of their complete plastome DNA sequences. Comparative analysis across plastomes in Melanthiaceae revealed that their structures and gene contents are highly conserved and provided molecular synapomorphies for some lineages of Parideae. Phylogenomic analysis and molecular dating recovered the evolutionary backbone of *Paris* and thus elucidated the phylogenetic position of *P. japonica*. The tree topologies and molecular dating indicated that the expansion of the ancestral genome of extant *Paris* and *Trillium* was probably a gradual process lasting for approximately 20 million years; the divergence of *P. japonica* and the section *Euthyra* may have been triggered by the opening of the Japan Sea, which separated the Japan Islands from the continent of Asia around the transition of the Oligocene/Miocene (20.30 Mya). This long-term separation would have played an important role in the formation and evolution of genomic gigantism in *P. japonica*. The phylogenetic position of *P. japonica* implies that the giant genomes of *Paris* and *Trillium* may have formed and evolved independently, even though the two genera are closely related. In addition, our phylogenomic analysis strongly supports the taxonomic treatment of *Paris* as a genus rather than dividing it into three genera, but did not support the recognition of *T. govanianum* as the separate genus *Trillidium*.

## Methods

### Plant material and shotgun sequencing

Leaf tissues of *P. japonica*, *P. verticillata*, *T. govanianum*, *Y. thibetica* and *Y. yunnanensis* were collected in the field and then dried with silica gel (one individual per species**)**. The vouchers were identified by Dr. Yunheng Ji and deposited at the herbarium of Kunming Institute of Botany, Chinese Academy of Sciences (KUN); the voucher information is presented in Table 3. Genomic DNA was extracted from *~* 20 mg of leaf tissue using a modified CTAB method [62]. Approximately 5 μg of purified genomic DNA was sheared by sonication. Paired-end libraries with an average insert size 350 bp were prepared using a TruSeq DNA Sample Prep Kit (Illumina, Inc., USA) according to the manufacturer’s protocol. Shotgun sequencing was performed on the Illumina HiSeq 2000 platform.

**Table 3:** Plastomes newly generated in this study with taxon, source, voucher information, and GenBank accessions

Species	Source of plant material	Voucher (Herbarium)	Genbank accession
*Paris japonica*	Chubu, Honshu, Japan	J. Maruta s. n. (KUN)	MF796668
*P. verticillata*	Jamusi, Heilongjiang, China	L. X. Wang s. n. (KUN)	MF796669
*Trillium govanianum*	Dingri, Tibet, China	S. K. Chen 1,289,634 (KUN)	MF796670
*Ypsilandra yunnanensis*	Gongshan, Yunnan, China	Y. Ji 2,007,014 (KUN)	MF796672
*Y. thibetica*	Nanchuan, Chongqing, China	Y. Ji 2,013,031 (KUN)	MF786671

### Plastome assembly, annotation and comparison

Raw Illumina reads were filtered by NGS QC tool kit [63] to remove adaptors and low-quality reads. The pipeline developed by Jin et al. [64] was used for de novo plastome assembly. The clean reads of *Paris* species, *T. govaniamum* and *Ypsilandra* species were mapped onto the reference plastomes of *P. quadrifolia* (Genbank accession: KX784051), *T. tschonoskii* (Genbank accession: KR780076) and *Heloniopsis tubiflora* (Genbank accession: KM078036) using the Bowtie v2.2.6 software [65] with its default parameters and preset options. All of the plastid-like reads were assembled into contigs by SPAdes v3.10.1 [66] with the *k*-mer defined as 75, 85, 95 and 105. A customized python script [64], which can use BLAST and a built-in library to search the plastid-like contig, was employed to connect verified contigs into plastomes in SPAdes v3.10.1 [66], with its default parameters. The results of de novo assembly were visualized and edited with Bandage v.8.0 [67].

The resulting plastomes were annotated by Dual Organellar Genome Annotator database [68]. The annotations were manually proofed using Geneious v10.2.3 [69]. The start and stop codons of protein-coding genes were checked manually. All of the identified tRNA was verified by tRNAscan-SE v1.21 [70], with the preset parameters. Functional classification of the plastid genes was determined by referring to the online database CpBase (http://rocaplab.ocean.washington.edu/old_website/tools/cpbase). The maps of plastomes were constructed with the Organellar Genome DRAW program [71].

The general features of plastome, such as structural rearrangements, gene loss/pseudogenization, gene duplication, and expansion/contraction of the IR regions, have provided evolutionary information in previous studies [15, 32, 72]. Therefore, we performed comparisons of these features among Melanthiaceae plastomes. The gene content and arrangement were visualized and compared with the MUMmer 3.0 program [73]. The boundaries of the LSC, IR, and SSC regions in each plastome were compared using Geneious v10.2.3 [69].

### Phylogenomic analysis

To examine the phylogenetic position of *P. japonica*, 24 plastomes representing wide phylogenetic diversity in the family Melanthiaceae were included in the phylogenomic analysis (Additional file 1: Table S1). The plastomes of *Campynema lineare*, *Fritillaria cirrhosa*, *Luzuriaga radicans* and *Smilax china* were used to root the tree. Of those, five plastomes were newly generated in the current study (Table 3), and the rest plastomes were downloaded from the NCBI database (Additional file 1: Table S).

The complete plastome DNA sequences were aligned using MAFFT [74] integrated in Geneious v.10.2.3 [69], with manual adjustment if necessary. The phylogenomic analyses were carried out with the standard Maximum Likelihood (ML) and Bayesian Inference (BI) methods. ML analyses were performed using RAxML-HPC BlackBox v8.1.24 [75] with 1000 replicates of rapid bootstrapping (BS) under the GTR-GAMMA model. The search of the best-scoring ML tree and rapid bootstrapping were performed in a single run. The choice of the best nucleotide sequence substitution model for BI analysis was determined using Modeltest v3.7 [51] with the Akaike Information Criterion [76]. BI was performed with MRBAYES v.3.1.2 [77] using the model (TVM + I + G) selected. Two independent parallel Markov Chain Monte Carlo (MCMC) runs with tree sampling every 100 generations for one million generations, with the first 25% discarded as burn-in, were conducted. Stationarity was considered to be reached when the average standard deviation of the split frequencies was < 0.01. The posterior probability values (PP) were determined from the remaining 0.75 million trees.

### Molecular dating

To date, no fossils have been identified for the family Melanthiaceae and its close relatives. Calibrated by 17 fossils across the monocots and major clades of angiosperms, a previous study [41] revealed that the crown age of family Melanthiaceae was approximately 84.8 Mya, while the clades Parideae-Xerophyllideae and Chionographideae-Heloniadeae diverged approximately 74 Mya, and the tribes Parideae and Xerophyllideae split approximately 52.3 Mya. We used these events to calibrate the phylogenetic tree (Fig. 1a).

Molecular dating was performed using the MCMCTREE v4.9c program integrated in the PAML program package [78]. The ML tree topology was used to estimate the divergence times of nodes. The independent-rates molecular clock was chosen as the clock model, and HKY85 was selected as the substitution model. The root age was set as less than 100 Mya. The divergence of Melanthiaceae was calibrated with a minimum age of 84.8 Mya. The node uniting Parideae-Xerophyllideae and Chionographideae-Heloniadeae was set to a minimum age of 74 Mya, while the divergence of Parideae and Xerophyllideae was set to a minimum age of 52.3 Mya. Other parameters were defined as their defaults. MCMC chains were run for 10,100,000 iterations. The first 100,000 iterations were discarded as burn-in, and trees were sampled every 10 iterations until 1000,000 samples were gathered.

## Additional files

Additional file 1: Table S1. Plastomes included in the phylogenetic analyses with GenBank accession. (DOCX 19 kb)

Additional file 2: **Table S2.** Summary of the Illumina sequencing results of *Paris japonica*, *P. verticillata*, *Trillium govanianum*, *Ypsilandra thibetica* and *Y. yunnanensis*. (DOCX 14 kb)

Additional file 3: **Table S3.** List of the genes identified in the plastome of Paris japonica. (DOCX 16 kb)

Additional file 4: **Table S4.** List of the genes identified in the plastome of *Paris verticillata*. (DOCX 16 kb)

Additional file 5: **Table S5.** List of the genes identified in the plastome of *Trillium govanianum*. (DOCX 16 kb)

Additional file 6: **Table S6.** List of the genes identified in the plastome of *Ypsilandra thibetica. (DOCX 16 kb)*

Additional file 7: **Table S7.** List of the genes identified in the plastome of *Ypsilandra yunnanensis*. (DOCX 16 kb)

Additional file 8: **Figure S1.** Map of the *Paris japonica* plastome. Genes shown outside the circle are transcribed clockwise, and those inside are transcribed counterclockwise. The dark grey area in the inner circle indicates the CG content of the plastome. (JPG 5223 kb)

Additional file 9: **Figure S2.** Map of the *Paris verticillata* plastome. Genes shown outside the circle are transcribed clockwise and those inside are transcribed counterclockwise. The dark grey area in the inner circle indicates the CG content of the plastome. (JPG 5226 kb)

Additional file 10: **Figure S3.** Map of the *Trillium govanianum* plastome. Genes shown outside the circle are transcribed clockwise, and those inside are transcribed counterclockwise. The dark grey area in the inner circle indicates the CG content of the plastome. (JPG 5223 kb)

Additional file 11: **Figure S4.** Map of the *Ypsilandra thibetica* plastome. Genes shown outside the circle are transcribed clockwise, and those inside are transcribed counterclockwise. The dark grey area in the inner circle indicates the CG content of the plastome. (JPG 5244 kb)

Additional file 12: **Figure S5.** Map of the *Ypsilandra yunnanensis* plastome. Genes shown outside the circle are transcribed clockwise, and those inside are transcribed counterclockwise. The dark grey area in the inner circle indicates the CG content of the plastome. (JPG 5287 kb)

## Data Availability

The complete cp genome sequences of *P. japonica*, *P. verticillata*, *Trillium govanianum*, *Ypsilandra thibetica* and *Y. yunnanensis* are available at GenBank under the accession numbers MF796668–MF796672. The data used in the analysis are included within the article and the additional files.

## Abbreviations

BI: Bayesian Inference
bp: Base pair
BS: Bootstrap
CTAB: Cetyl trimethylammonium bromide
HPD: Highest posterior density
IR: Inverted repeat
ITS: internal transcribed spacer of nuclear ribosomal DNA
MCMC: Markov Chain Monte Carlo
ML: Maximum Likelihood
Mya: Million years ago
NCBI: National Center for Biotechnology Information
rRNA: Ribosomal RNA
SSC: Small single copy
tRNA: Transfer RNA
LSC: Large single-copy

## References

[1] Pellicer J, Fay MF, Leitch IJ (2010). The largest eukaryotic genome of them all?. Bot J Linn Soc.

[2] Pellicer J, Kelly LJ, Magdalena C, Leitch IJ (2013). Insight into the dynamics of genome size and chromosome evolution in the early diverging angiosperm lineage Nymphaeales (water lilies). Genome..

[3] Fedoroff NV (2012). Transposable elements, epigenetics, and genome evolution. Science..

[4] Dodsworth S, Leitch AR, Leitch IJ (2015). Genome size diversity in angiosperms and its influence on gene space. Curr Opin Genet Dev.

[5] Leitch IJ, Chase MW, Bennett MD (1998). Phylogenetic analysis of DNA C-values provides evidence for a small ancestral genome size in flowering plants. Ann Bot.

[6] Leitch IJ, Beaulieu JM, Chase MW, Leitch AR, Fay MF (2010). Genome size dynamics and evolution in monocots. J Bot.

[7] Zonneveld BJM, Leitch IJ, Bennett MD (2005). First nuclear DNA amounts in more than 300 angiosperms. Ann Bot.

[8] Leitch Ilia J., Leitch Andrew R. (2012). Genome Size Diversity and Evolution in Land Plants. Plant Genome Diversity Volume 2.

[9] Hidalgo O, Pellicer J, Christenhusz M, Schneider H, Leitch AR, Leitch IJ (2017). Is there an upper limit to genome size?. Trends Plant Sci.

[10] Bennetzen JL, Ma JX, Devos K (2005). Mechanisms of recent genome size variation in flowering plants. Ann Bot.

[11] Morgan MT (2001). Transposable element number in mixed mating populations. Genet Res.

[12] Wendel JF, Cronn RC, Johnston JS, Price HJ (2002). Feast and famine in plant genomes. Genetica..

[13] Farmer SB, Schilling EE (2002). Phylogenetic analyses of Trilliaceae based on morphological and molecular data. Syst Bot.

[14] Murray RV, Nickrent DC (2004). A molecular phylogeny of the mistletoe genus *Viscum*. Trans Illin State Acad Sci.

[15] Huang Y, Li X, Yang Z, Yang C, Yang J, Ji Y (2016). Analysis of complete chloroplast genome sequences improves phylogenetic resolution in *Paris* (Melanthiaceae). Front Plant Sci.

[16] Gregory TR, Hebert PD (1999). The modulation of DNA content: proximate causes and ultimate consequences. Genome Res.

[17] Hidalgo O, Pellicer J, Christenhusz MJM, Schneider H, Leitch IJ (2017). Genomic gigantism in the whisk-fern family (Psilotaceae): *Tmesipteris obliqua* challenges record holder *Paris japonica*. Bot J Linn Soc.

[18] Zomlefer WB, Williams NH, Whitten WM, Judd WS (2001). Generic circumscription and relationships in the tribe Melanthieae (Liliales, Melanthiaceae), with emphasis on *Zigadenus*: evidence from ITS and *trnL-F* sequence data. Am J Bot.

[19] Angiosperm Phylogeny Group (2016). An update of the angiosperm phylogeny group classification for the orders and families of flowering plants: APG IV. Bot J Linn Soc.

[20] Takewaki M, Sutô T (1935). On the new genus kinugasa. Trans Sapporo Not Hist Soc.

[21] Li H, Li H (1998). The phylogeny of the genus *Paris* L. The genus Paris L.

[22] Haga T (1938). Chromosome complement of *Kinugasa japonica* with special reference to its origin and hebavior. Cytologia..

[23] Hara H (1969). Variations in *Paris polyphylla* smith, with reference to other Asiatic species. J Fac Sci Univ Tokyo Sect III.

[24] Tahktajan A (1983). A revision of *Daiswa* (Trilliaceae). Brittonia..

[25] Kato H, Terauchi R, Utech FH, Kawano S (1995). Molecular systematics of the Trilliaceae *sensu lato* as inferred from *rbc*L sequence data. Mol Phylogenet Evol.

[26] Osaloo SK, Kawano S (1999). Molecular systematics of Trilliaceae II. Phylogenetic analyses of *Trillium* and its allies using sequences of *rbc*L and *mat*K genes of cpDNA and internal transcribed spacers of 18S–26S nrDNA. Plant Species Biol.

[27] Ji Y, Fritsch PW, Li H, Xiao T, Zhou Z (2006). Phylogeny and classification of *Paris* (Melanthiaceae) inferred from DNA sequence data. Ann Bot.

[28] Kim S, Kim JS, Chase MW, Chase MW, Fay MF, Kim J (2016). Molecular phylogenetic relationships of Melanthiaceae (Liliales) based on plastid DNA sequences. Bot J Linn Soc.

[29] Rokas A, Carroll SB (2005). More genes or more taxa? The relative contribution of gene number and taxon number to phylogenetic accuracy. Mol Biol Evol.

[30] Philippe H, Brinkmann H, Lavrov DV, Littlewood DTJ, Manuel M, Wörheide G (2011). Resolving difficult phylogenetic questions: why more sequences are not enough. PLoS Biol.

[31] Parks M, Cronn R, Liston A (2009). Increasing phylogenetic resolution at low taxonomic levels using massively parallel sequencing of chloroplast genomes. BMC Biol.

[32] Jansen RK, Cai Z, Raubeson LA, Daniell H, Leebens-Mack J, Müller KF, Guisinger-Bellian M (2007). Analysis of 81 genes from 64 plastid genomes resolves relationships in angiosperms and identifies genome-scale evolutionary patterns. Proc Natl Acad Sci U S A.

[33] Moore MJ, Bell CD, Soltis PS, Soltis DE (2007). Using plastid genome-scale data to resolve enigmatic relationships among basal angiosperms. Proc Natl Acad Sci U S A.

[34] Moore MJ, Soltis PS, Bell CD, Burleigh JG, Soltis DE (2010). Phylogenetic analysis of 83 plastid genes further resolves the early diversification of eudicots. Proc Natl Acad Sci U S A.

[35] Barrett CF, Specht CD, Leebens-Mack J, Stevenson DW, Zomlefer WB, Davis JI (2014). Resolving ancient radiations: can complete plastid gene sets elucidate deep relationships among the tropical gingers (Zingiberales)?. Ann Bot.

[36] Stull GW, Dunod SR, Soltis DE, Soltis PS (2015). Resolving basal lamiid phylogeny and the circumscription of Icacinaceae with a plastome-scale data set. Am J Bot.

[37] Attigala L, Wysocki WP, Duvall MR, Clark LG. Phylogenetic estimation and morphological evolution of Arundinarieae (Bambusoideae: Poaceae) based on plastome phylogenomic analysis. Mol Phylogen Evol. 2016; 101: 111–121. doi: org/10.1016/j.ympev.2016.05.008. 10.1016/j.ympev.2016.05.008 27164472

[38] Straub SC, Parks M, Weitemier K, Fishbein M, Cronn RC, Liston A (2012). Navigating the tip of the genomic iceberg: next-generation sequencing for plant systematics. Am J Bot.

[39] Kim JS, Hong JK, Chase MW, Fay MF, Kim JH (2013). Familial relationships of the monocot order Liliales based on a molecular phylogenetic analysis using four plastid loci: *matK*, *rbcL*, *atpB* and *atpF-H*. Bot J Linn Soc.

[40] Pellicer J, Kelly LJ, Leitch IJ, Zomlefer WB, Fay MF (2014). A universe of dwarfs and giants: genome size and chromosome evolution in the monocot family Melanthiaceae. New Phytol.

[41] Givnish TJ, Zuluaga A, Marques I, Lam VKY, Gomez MS, Iles WJD (2016). Phylogenomics and historical biogeography of the monocot order liliales: out of Australia and through Antarctica. Cladistics..

[42] Millen RS, Olmstead RG, Adams KL, Palmer JD, Lao NT, Heggie L (2001). Many parallel losses of infA from chloroplast DNA during angiosperm evolution with multiple independent transfers to the nucleus. Plant Cell.

[43] Wolfe KH, Morden CW, Palmer JD (1992). Function and evolution of a minimal plastid genome from a nonphotosynthetic parasitic plant. Proc Natl Acad Sci U S A.

[44] Logacheva MD, Schelkunov M, Penin AA (2011). Sequencing and analysis of plastid genome in mycoheterotrophic orchid Neottia nidus-avis. Genome Biol Evol..

[45] Wicke S, Müller KF, Depamphilis CW, Quandt D, Bellot S, Schneeweiss GM (2016). Mechanistic model of evolutionary rate variation en route to a nonphotosynthetic lifestyle in plants. Proc Natl Acad Sci U S A.

[46] Willey DL, Gray JC (1990). An open reading frame encoding aputativehaem-binding polypeptide is cotranscribed with the pea chloroplast gene for apocytochrome f. Plant Mol Biol.

[47] Do HDK, Kim JS, Kim JH (2014). A trnI_CAU triplication event in the complete chloroplast genome of *Paris verticillata* M. Bieb. (Melanthiaceae, Liliales). Genome Biol Evol.

[48] Wang RJ, Cheng CL, Chang CC, Wu CL, Su TM, Chaw SM (2008). Dynamics and evolution of the inverted repeat-large single copy junctions in the chloroplast genomes of monocots. BMC Evol Biol.

[49] Zhu A, Guo W, Gupta S, Fan W, Mower JP (2016). Evolutionary dynamics of the plastid inverted repeat: the effects of expansion, contraction, and loss on substitution rates. New Phytol.

[50] Lopes ADS, Pacheco TG, Nimz T, Vieira LDN, Guerra MP, Nodari RO (2018). The complete plastome of macaw palm [ Acrocomia aculeata, (jacq.) lodd. Ex mart.] and extensive molecular analyses of the evolution of plastid genes in Arecaceae. Planta..

[51] Posada D, Crandall KA (1998). MODELTEST: testing the model of DNA substitution. Bioinformatics..

[52] Hara H, Stearn WT, Williams LHJ. An enumeration of the flowering plants of Nepal. Taxon. 1978; III: 384.

[53] Santosh M (2011). History of supercontinents and its relation to the origin of Japanese Islands. J Geodyn.

[54] Kang M, Tao J, Wang J, Ren C, Qi Q, Xiang QY (2014). Adaptive and nonadaptive genome size evolution in karst endemic flora of China. New Phytol.

[55] Wright NA, Gregory TR, Witt CC (2014). Metabolic ‘engines’ of flight drive genome size reduction in birds. Proc Biol Sci.

[56] Carta A, Puruzzi L (2016). Testing the large genome constraint hypothesis: plant traits, habitat and climate seasonality in Liliaceae. New Phytol.

[57] Knight CA, Molinari N, Petrov D (2005). The large genome constraint hypothesis: evolution, ecology, and phenotype. Ann Bot.

[58] Knight CA, Beaulieu JM (2008). Genome size scaling through phenotype space. Ann Bot.

[59] Wan SM, Li AC, Clift PD, Stut JBW (2007). Development of the east Asian monsoon: mineralogical and sedimentologic records in the northern South China Sea since 20 Ma. Palaeogeogr Palaeoclimatol Palaeoecol.

[60] Jacques FM, Guo SX, Su T, Xing YW, Huang YJ, Liu YS (2011). Quantitative reconstruction of the Late Miocene monsoon climates of Southwest China: a case study of the Lincang flora from Yunnan Province. Palaeogeogr Palaeoclimatol Palaeoecol.

[61] Fukutome S, Frei C, Schär C (2003). The influence of SST on the interannual variability of Japan's summer precipitation. J Meteorol Soc Jap.

[62] Doyle JJ, Doyle JL (1987). A rapid DNA isolation procedure for small quantities of fresh leaf tissue. Phytochem Bull.

[63] Patel RK, Jain M (2012). NGS QC toolkit: a toolkit for quality control of next generation sequencing data. PLoS One.

[64] Jin JJ, Yu WB, Yang JB, Song Y, Yi TS, Li DZ. GetOrganelle: a simple and fast pipeline for de novo assembly of a complete circular chloroplast genome using genome skimming data. bioRxiv. 2018; 256479. doi: 10.1101/256479

[65] Bankevich A, Nurk S, Antipov D, Gurevich AA, Dvorkin M, Kulikov AS (2012). SPAdes: a new genome assembly algorithm and its applications to single-cell sequencing. J Comp Biol.

[66] Langmead B, Salzberg SL (2012). Fast gapped-read alignment with Bowtie2. Nat Methods.

[67] Wick RR, Schultz MB, Zobel J, Holt KE (2015). Bandage: interactive visualization of de novo genome assemblies. Bioinformatics..

[68] Wyman SK, Jansen RK, Boore JL (2004). Automatic annotation of organellar genomes with DOGMA. Bioinformatics..

[69] Kearse M, Moir R, Wilson A, Stoneshavas S, Cheung M, Sturrock S (2012). Geneious basic: an integrated and extendable desktop software platform for the organization and analysis of sequence data. Bioinformatics..

[70] Schattner P, Brooks AN, Lowe TM (2005). The tRNAscan-SE, snoscan and snoGPS web servers for the detection of tRNAs and snoRNAs. Nucl Acids Res.

[71] Lohse M, Drechsel O, Bock R (2007). OrganellarGenomeDRAW (OGDRAW): a tool for the easy generation of high-quality custom graphical maps of plastid and mitochondrial genomes. Curr Genet.

[72] Raubeson LA, Peery R, Chumley TW, Dziubek C, Fourcade HM, Boore JL (2007). Comparative chloroplast genomics: analyses including new sequences from the angiosperms *Nupha radvena* and *Ranunculus macranthus*. BMC Genomics.

[73] Kurtz S, Phillippy A, Delcher AL, Smoot M, Shumway M, Antonescu C (2004). Versatile and open software for comparing large genomes. Genome Biol.

[74] Katoh K, Standley DM (2013). MAFFT multiple sequence alignment software version 7: improvements in performance and usability. Mol Biol Evol.

[75] Stamatakis A (2006). RAxML-VI-HPC: maximum likelihood-based phylogenetic analysis with thousands of taxa and mixed models. Bioinformatics..

[76] Posada D, Buckley TR (2004). Model selection and model averaging in phylogenetics: advantages of Akaike information criterion and Bayesian approaches over likelihood ratio tests. Syst Biol.

[77] Ronquist F, Huelsenbeck JP (2003). MrBayes 3: Bayesian phylogenetic inference under mixed models. Bioinformatics..

[78] Yang Z (1997). PAML: a program package for phylogenetic analysis by maximum likelihood. CABIOS.

