# Supplementary material for: Chloroplast phylogenomic analysis provides insights into the evolution of the largest eukaryotic genome holder, Paris japonica (Melanthiaceae)
Source: BMC Plant Biol. 2019 Jul 4;19:293. doi: 10.1186/s12870-019-1879-7 (PMC6611055; doi:10.1186/s12870-019-1879-7)
Supplement: Supplementary file 1 — Table S1. Plastomes included in the phylogenetic analyses with GenBank accession. (DOCX 19 kb) [file 12870_2019_1879_MOESM1_ESM.docx]

**Table S1.** Plastomes included in phylogenetic analyses with GenBank accession.

| *Taxon* | Plastome size (bp) | Order | Family | GenBank Accession |
| --- | --- | --- | --- | --- |
| *Paris cronquistii* | 156,896 | Liliales | [Melanthiaceae](https://en.wikipedia.org/wiki/Melanthiaceae) | KX784041 |
| *Paris dunniana* | 157,174 | Liliales | [Melanthiaceae](https://en.wikipedia.org/wiki/Melanthiaceae) | KX784042 |
| *Paris fargestii* | 157,518 | Liliales | [Melanthiaceae](https://en.wikipedia.org/wiki/Melanthiaceae) | KX784043 |
| *Paris forrestii* | 157,533 | Liliales | [Melanthiaceae](https://en.wikipedia.org/wiki/Melanthiaceae) | KX784044 |
| *Paris luquanensis* | 157,594 | Liliales | [Melanthiaceae](https://en.wikipedia.org/wiki/Melanthiaceae) | KX784045 |
| *Paris mairei* | 157,079 | Liliales | [Melanthiaceae](https://en.wikipedia.org/wiki/Melanthiaceae) | KX784046 |
| *Paris marmorata* | 156,755 | Liliales | [Melanthiaceae](https://en.wikipedia.org/wiki/Melanthiaceae) | KX784047 |
| *Paris polyphylla* var. *chinensis* | 157,847 | Liliales | [Melanthiaceae](https://en.wikipedia.org/wiki/Melanthiaceae) | KX784048 |
| *Paris polyphylla* var. *yunnanensis* | 156,737 | Liliales | [Melanthiaceae](https://en.wikipedia.org/wiki/Melanthiaceae) | KX784049 |
| *Paris vietnamensis* | 158,224 | Liliales | [Melanthiaceae](https://en.wikipedia.org/wiki/Melanthiaceae) | KX784050 |
| *Paris quadrifolia* | 157,907 | Liliales | [Melanthiaceae](https://en.wikipedia.org/wiki/Melanthiaceae) | KX784051 |
| *Paris japonica* | 155,957 | Liliales | [Melanthiaceae](https://en.wikipedia.org/wiki/Melanthiaceae) | MH796668 |
| *Paris verticillata* | 157,946 | Liliales | [Melanthiaceae](https://en.wikipedia.org/wiki/Melanthiaceae) | MH796669 |
| *Trillium govanianum* | 157,379 | Liliales | [Melanthiaceae](https://en.wikipedia.org/wiki/Melanthiaceae) | MH796670 |
| *Trillium cuneatum* | 156,610 | Liliales | [Melanthiaceae](https://en.wikipedia.org/wiki/Melanthiaceae) | KR135077 |
| *Trillium decumbens* | 158,552 | Liliales | [Melanthiaceae](https://en.wikipedia.org/wiki/Melanthiaceae) | KR534612 |
| *Trillium maculatum* | 157,359 | Liliales | [Melanthiaceae](https://en.wikipedia.org/wiki/Melanthiaceae) | KR780075 |
| *Trillium tschonoskii* | 156,852 | Liliales | [Melanthiaceae](https://en.wikipedia.org/wiki/Melanthiaceae) | KR780076 |
| *Veratrum patulum* | 153,699 | Liliales | [Melanthiaceae](https://en.wikipedia.org/wiki/Melanthiaceae) | KF437397 |
| *Chionographis japonica* | 154,646 | Liliales | [Melanthiaceae](https://en.wikipedia.org/wiki/Melanthiaceae) | KF951065 |
| *Ypsilandra yunnanensis* | 158,806 | Liliales | [Melanthiaceae](https://en.wikipedia.org/wiki/Melanthiaceae) | MH796672 |
| *Ypsilandra thibetica* | 157,612 | Liliales | [Melanthiaceae](https://en.wikipedia.org/wiki/Melanthiaceae) | MH796671 |
| *Heloniopsis tubiflora* | 158,229 | Liliales | [Melanthiaceae](https://en.wikipedia.org/wiki/Melanthiaceae) | KM078036 |
| *Xerophyllum tenax* | 156,746 | Liliales | [Melanthiaceae](https://en.wikipedia.org/wiki/Melanthiaceae) | KM078035 |
| *Campynema lineare* (outgroup) | 156,261 | Liliales | Campynemataceae | KP462881 |
| *Fritillaria cirrhosa* (outgroup) | 151,991 | Liliales | Liliaceae | KF769143 |
| *Luzuriaga radicans* (outgroup) | 157,885 | Liliales | Alstroemeriaceae | KM233640 |
| *Smilax china* (outgroup) | 157,878 | Liliales | Smilacaceae | HM536959 |
