# Supplementary material for: Chloroplast phylogenomic analysis provides insights into the evolution of the largest eukaryotic genome holder, Paris japonica (Melanthiaceae)
Source: BMC Plant Biol. 2019 Jul 4;19:293. doi: 10.1186/s12870-019-1879-7 (PMC6611055; doi:10.1186/s12870-019-1879-7)
Supplement: Supplementary file 2 — Table S2. Summary of the Illumina sequencing results of Paris japonica, P. verticillata, Trillium govanianum, Ypsilandra thibetica and Y. yunnanensis. (DOCX 14 kb) [file 12870_2019_1879_MOESM2_ESM.docx]

**Table S2.** Summary of Illumina sequencing of *Paris japonica*, *P. verticillata*, *Trillium govanianum*, *Ypsilandra thibetica* and *Y. yunnanensis*.

| Species | No. of clean  reads | Plastid-like reads  (percentage) | Sequencing  coverage (X) |
| --- | --- | --- | --- |
| *Paris japonica* | 25,745,358 | 408,201 (1.59%) | 387 |
| *P. verticillata* | 21,974,100 | 298,612 (1.36%) | 283 |
| *Trillium govanianum* | 28,448,452 | 633,367 (2.23%) | 600 |
| *Ypsilandra yunnanensis* | 30,925,986 | 1,146,491 (3.71%) | 1,086 |
| *Y. thibetica* | 25,443,172 | 939,421 (3.69%) | 842 |
