# Supplementary material for: Chloroplast phylogenomic analysis provides insights into the evolution of the largest eukaryotic genome holder, Paris japonica (Melanthiaceae)
Source: BMC Plant Biol. 2019 Jul 4;19:293. doi: 10.1186/s12870-019-1879-7 (PMC6611055; doi:10.1186/s12870-019-1879-7)
Supplement: Supplementary file 4 — Table S4. List of the genes identified in the plastome of Paris verticillata. (DOCX 16 kb) [file 12870_2019_1879_MOESM4_ESM.docx]

**Table S4.** List of genes identified in the plastome of [*Paris*](https://en.wikipedia.org/wiki/Melanthiaceae) *verticillata*.

| **Category of Genes** | **Group of gene** | **Name of gene** |
| --- | --- | --- |
| Self-replication | Ribosomal RNA genes | *rrn*4.5×2*,* *rrn*5×2*,* *rrn*16×2*, rrn*23×2 |
|  | Transfer RNA genes | *trn*C-GCA*, trn*D-GUC*, trn*E-UUC*, trn*F-GAA*, trn*G-GCC*, trn*G-UCC**, trn*H-GUG×2*, trn*K-UUU**, trn*L-UAA**, trn*L-UAG×2*, trn*M-CAU*, trn*P-UGG*, trn*Q-UUG*, trn*R-UCU*, trn*S-GCU*, trn*S-GGA*, trn*S-UGA*, trn*T-UGU*, trn*T-GGU*, trn*V-UAC**, trn*Y-GUA*, trn*W-CCA*, trn*fM-CAU*, trn*A-UGC* ×2*, trn*I-CAU*×*6*, trn*I-GAU*×2*, trn*L-CAA×2*, trn*N-GUU×2*,* *trn*R-ACG×2*, trn*V-GAC×2 |
|  | Ribosomal protein  (small subunit) | *rps*2*, rps*3*, rps*4*, rps*7×2*, rps*8*, rps*11*, rps*12**×2*, rps*14*, rps*15*, rps*16**, rps*18*, rps*19×2 |
|  | Ribosomal protein  (large subunit) | *rpl*2*×2*, rpl*14*, rpl*16**, rpl*20*, rpl*22×2*, rpl*23×2*, rpl*32*, rpl*33*, rpl*36 |
|  | RNA polymerase | *rpo*A*, rpo*B*, rpo*C1**, rpo*C2 |
|  | Translational initiation factor | *inf*A |
| Genes for photosynthesis | Subunits of photosystem I | *psa*A*, psa*B*, psa*C*, psa*I*，psa*J*, ycf*3***, ycf*4 |
|  | Subunits of photosystem II | *psb*A*, psb*B*, psb*C*, psb*D*, psb*E*, psb*F*, psb*H*, psb*I*, psb*J*, psb*K*, psb*L*, psb*M*, psb*N*, psb*T*, psb*Z |
|  | Subunits of cytochrome | *pet*A*, pet*B**, pet*D**, pet*G*, pet*L*, pet*N |
|  | Subunits of ATP synthase | *atp*A*, atp*B*, atp*E*, atp*F**, atp*H*, atp*I |
|  | Large subunit of Rubisco | *rbc*L |
|  | Subunits of NADH  dehydrogenase | *ndh*A**, ndh*B*×2*, ndh*C*, ndh*D*, ndh*E*, ndh*F*, ndh*G*, ndh*H*, ndh*I*, ndh*J*, ndh*K |
| Other genes | Maturase | *mat*K |
|  | Envelope membrane protein | *cem*A^#^ |
|  | Subunit of acetyl-CoA | *acc*D |
|  | Synthesis gene | *ccs*A |
|  | ATP-dependent protease | *clp*P**** |
|  | Component of TIC complex | *ycf*1 |
| Genes of  unknown function | Conserved open reading frames | *ycf*2×2*, ycf*15^#^×2 |

×2: Two gene copies in IR regions; *: With one intron; **: With two introns; ^#^: Pseudogene.
